# Clinical and laboratory findings associated with severe scrub typhus

**DOI:** 10.1186/1471-2334-10-108

**Published:** 2010-04-30

**Authors:** Dong-Min Kim, Seok Won Kim, Seong-Hyung Choi, Na Ra Yun

**Affiliations:** 1 Division of Infectious Diseases, Departments of Internal Medicine, Chosun University School of Medicine, Gwangju City, Republic of Korea; 2 Department of Neurosurgery, Chosun University School of Medicine, Gwangju City, Republic of Korea; 3 Research Center for Resistant Cell, Chosun University, School of Medicine, Gwangju City, Republic of Korea

## Abstract

**Background:**

Scrub typhus is a mite-borne bacterial infection of humans caused by *Orientia tsutsugamushi *that causes a generalized vasculitis that may involve the tissues of any organ system. The aim of this study was to identify factors associated to severe complications from scrub typhus.

**Methods:**

We conducted this prospective, case-control study on scrub typhus patients who presented to the Department of Internal Medicine at Chosun University Hospital between September, 2004 and December, 2006. Cases were 89 scrub typhus patients with severe complications and controls were 119 scrub typhus patients without severe complications.

**Results:**

There were significant differences in the absence of eschar, white blood cell (WBC) counts, hemoglobin, albumin, serum creatinine, fibrinogen, C-reactive protein (CRP), and active partial thromboplastin time (aPTT) between the two groups. Multivariate analysis demonstrated that only the following four factors were significantly associated with the severe complications of scrub typhus: (1) age ≥ 60 years (odd ratio [OR] = 3.13, *P *= 0.002, confidence interval [CI] = 1.53-6.41), (2) the absence of eschar (OR = 6.62, *P *= 0.03, CI = 1.22-35.8, (3) WBC counts > 10, 000/mm3 (OR = 3.6, *P *= 0.001, CI = 1.65-7.89), and (4) albumin ≤ 3.0 g/dL (OR = 5.01, *P *= 0.004, CI = 1.69-14.86).

**Conclusions:**

Our results suggest that clinicians should be aware of the potential for complications, when scrub typhus patients are older (≥ 60 years), presents without eschar, or laboratory findings such as WBC counts > 10, 000/mm3, and serum albumin level ≤ 3.0 g/dL. Close observation and intensive care for scrub typhus patients with the potential for complications may prevent serious complications with subsequent reduction in its mortality rate.

## Background

Scrub typhus is a mite-borne bacterial infection of humans caused by *Orientia tsutsugamushi *that leads to generalized vasculitis which may involve the tissues of any organ systems [1,2]. Reported severe clinical manifestations or complications of scrub typhus include interstitial pneumonia, acute renal failure, meningoencephalitis, gastrointestinal bleeding, and multiple organ failures. Since patients with scrub typhus may die from such complications, we should pay close attention to this disease entity [3-6]. Most patients with scrub typhus can recover without specific complications with early diagnosis and management. Its early clinical manifestations are nonspecific and are characterized by fever, chill, headache, and myalgia. Recent studies on the clinical characteristics of scrub typhus have reported many abnormal laboratory findings [2,7]. However, there have been a few studies on the markers for the severity of scrub typhus [8]. It is necessary to determine the predictors that identify markers of severe disease in order to reduce the mortality due to the high incidence of severe manifestations or complications and the delay in treatment. Based on the markers, severe disease-prone patients can be admitted earlier to a hospital or transferred to a well-equipped institution. Close observation and intensive care can prevent complications and severe morbidity or mortality. Therefore, this study was conducted to identify the markers associated with severe scrub typhus.

## Methods

Between September 1, 2004 and December 31, 2006, we conducted a prospective study on patients suffering with possible scrub typhus infections who presented to the Department of Internal Medicine at Chosun University Hospital located in the southwestern part of Korea with acute febrile diseases. Of the patients, those aged ≥ 18 years who showed eschar or maculopapular skin rashes or were diagnosed clinically as having possible scrub typhus by the specialist in infectious diseases were enrolled for the study. The diagnosis of scrub typhus was confirmed when an indirect immunofluorescent antibody assay (IFA) IgM titer against *O. tsutsugamushi *increased to ≥ 1:80 or an IFA titer against *O. tsutsugamushi *increased four times or more [9].

Severe scrub typhus was defined if it showed the following conditions: (1) pneumonia that revealed parenchymal lung lesions on chest radiographs and cough or dyspnea, (2) renal failure that revealed estimated creatinine clearance less than 50 using the Cockcroft-Gault formula [10], (3) meningoencephalitis that exhibited altered mental states such as confusion, obtundation, stupor, or coma without evident causes such as shock or hypoglycemia, or the presence of both severe headaches and neck stiffness, or cerebrospinal fluid (CSF) counts of ≥ 5 leukocytes/mm^3^, (4) shock, which is defined by a systolic blood pressure less than 90 mmHg (or a fall in systolic blood pressure of > 40 mmHg), (5) myocarditis, (6) GI bleeding, or (7) death.

The confirmed scrub typhus patients were divided into two groups: a case group with severe scrub typhus and a control group without severe scrub typhus.

At presentation, a thorough history taking, physical examination, and hematologic laboratory tests were performed in the patients who were enrolled in this study. In addition, scrub typhus-like diseases, including murine typhus, leptospirosis, epidemic hemorrhagic fever, and systemic lupus erythematosus were excluded based on the laboratory tests and clinical features. Signed informed consent was obtained for each patient before the patients were included in the study. This study was approved by the Institutional Review Board of our hospital.

The collected data were stored using the computer program. Continuous data are expressed as mean ± SD, and the comparisons of the means between the two study groups were made using the unpaired *t *test. Nominal data are expressed as frequency or proportion, and the chi square test and the Fisher's exact test were applied to compare the differences in proportion between the two study groups. The associations between markers and severe scrub typhus were determined using the chi square test and the univariate logistic regression analysis. Input variables for the multivariate analysis were selected from significant variables obtained from the univariate analysis. In order to determine the markers related to the occurrence of severe scrub typhus, the multivariate logistic regression analysis was performed with the independent variables being those significantly associated with severe scrub typhus in the univariate analysis as independent variables. The linearity of continuous variables was explored and dichotomized using the mean as the cutoff point before the model fitting. The model was fitted using both backward and forward model selection methods. The odds ratio with 95% confidence intervals are presented to examine the statistical significance. Statistical analyses were performed using the SPSS software, version 12.0 (SPSS Inc, Chicago, IL).

## Results and Discussion

A total of 333 patients fulfilled the criteria for possible scrub typhus infection during the study period. Of those, 87 patients did not meet the inclusion criteria (44 patients refused to enroll in our study, 40 patients were confirmed as disease other than scrub typhus, and 3 patients had concurrent infection with hemorrhagic fever with renal syndrome). We could not confirm as scrub typhus in 38 patients due to failure to demonstrate a fourfold or greater rise in antibody titer. Hence, a total of 208 patients were finally confirmed to have scrub typhus by serology.

Severe scrub typhus such as pneumonia, renal failure, meningoencephalitis, shock, gastrointestinal bleeding, myocarditis, and death was observed in 89 of 208 (42.8%) patients with a definitive diagnosis of scrub typhus. This content is summarized in table 1. Patients were divided into two groups: case group with severe scrub typhus (n = 89) and control group without severe scrub typhus (n = 119).

**Table 1:** Distribution of severe scrub typhus cases

Complications	No. (%)
Pneumonia	53 (25.5%)
Renal failure	32 (15.4%)
Meningoencephalitis	23 (11.1%)
Shock	15 (7.2%)
Gastrointestinal bleeding	6 (2.9%)
Myocarditis	5 (2.4%)
Death	1 (0.5%)

Presenting symptoms and signs and laboratory findings at the initial visit are summarized in Table 2. The mean age of the case group was 68.56 ± 11.1 years, which was greater than that of the control group. There was no significant difference in duration from disease onset to effective antibiotics (such as tetracyclines, macrolides, and rifamycins) between the cases and the controls. However, there were significant differences in the presence of eschar, WBC counts, hemoglobin, albumin, serum creatinine, fibrinogen, C-reactive protein (CRP), and active partial thromboplastin time (aPTT) between the two groups (Table 2); Many confidence intervals in variables of Table 2, 3 are relatively wide. The linearity of continuous variables was dichotomized using the mean as the cutoff point, and the univariate logistic regression model was fitted to test for correlations between the severe scrub typhus and the seven factors, showing that there were significant correlations (Table 3). The seven factors were as follows: (1) age ≥ 60 years, (2) the absence of eschar, (3) WBC counts > 10, 000/mm^3^, (4) hemoglobin ≤ 10 g/dL, (5) albumin ≤ 3.0 g/dL, (6) serum creatinine > 1.4 mg/dL, and (7) CRP > 10 mg/dL. Since the serum creatinine level was included in the definition of severe scrub typhus, six factors except this one were included in the multivariate model for severe scrub typhus. Since both backward and forward model selection methods produced similar results, data from the forward selection model are presented.

**Table 2:** Demographic, clinical characteristics and laboratory findings on admission of the 208 scrub typhus patients

Characteristics	Severe scrub typhus (n = 89)	Non-severe scrub typhus (n = 119)
Demographic data		
Age, mean years ± SD*	68.56 ± 11.1	57.32 ± 15.7
Gender, no. of male/no. of female (% of male)	28/61(31.5)	43/76(36.1)
Duration of illness before admission, days	8.13 ± 5.4	6.95 ± 3.7
Length of hospitalization, mean days ± SD*	11.5 ± 9.6	7.21 ± 2.8
Mean duration from disease onset to effective antibiotic therapy (days)	8.34 ± 5.1	7.11 ± 3.7
Underlying diseases (Diabetes, Liver cirrhosis, COPD)	6(6.8%)	4(3.3%)
Clinical symptoms and signs		
Headache, no. (%) of patients	74 (83.1)	109 (91.6)
Myalgia, no. (%) of patients	66 (74.2)	95(79.8)
Skin rash, no. (%) of patients	78(87.6)	111 (93.3)
Eschar, no.(%) of patients*	77 (86.5)	117 (98.3)
Laboratory values		
WBC count (no. of cells × 1, 000/mm^3^) *	9.4 ± 4.3	6.6 ± 3.1
Hemoglobin (g/dL) *	12.0 ± 1.8	12.7 ± 1.3
Platelet count (no. of cells × 1, 000/mm^3^)	149.31 ± 77.3	153.7 ± 72.9
AST (IU/L)	100.9 ± 70.0	104.1 ± 86.8
ALT (IU/L)	75.0 ± 74.4	91.2 ± 86.5
ALP (U/L)	136.4 ± 120.0	118.5 ± 87.1
Bilirubin (mg/dL)	1.0 ± 1.0	0.8 ± 0.6
Albumin (g/dL) *	3.3 ± 0.6	3.8 ± 0.5
LDH (U/L)	818.5 ± 240.1	874.3 ± 264.2
CPK (U/L)	232.3 ± 609.5	263.5 ± 698.5
ADA (IU/L)	83.6 ± 24.9	79.6 ± 24.7
Serum creatinine (mg/dL) *	1.5 ± 0.9	1.0 ± 0.2
Fibrinogen (mg/dL) *	309.2 ± 97.4	348.2 ± 90.4
CRP (mg/dL) *	10.5 ± 5.0	8.1 ± 5.6
ESR (mm/hr)	19.4 ± 17.7	7.0 ± 15.0
PT (sec)	12.2 ± 1.0	12.3 ± 1.3
aPTT (sec) *	32.6 ± 5.8	30.5 ± 4.6

Values are mean ± SD.

* There was significant difference in baseline characteristics between the two groups (*P *< .05).

**AST **= aspartate aminotransferase; **ALT **= alanine aminotransferase; **LDH **= Lactate dehydrogenase; **CPK **= Creatine Kinase; **ADA **= adenosine deaminase; **CRP **= C-reactive protein; COPD = Chronic obstructive pulmonary disease

**Table 3:** Unadjusted relative risk for each selected factor on severe scrub typhus

Factors	Severe scrub typhus (n = 89)	Non-severe scrub typhus (n = 119)	Odds ratio (CI)	*P *value
Age(year) > 60	71 (79.8%)	57 (47.9%)	4.3 (2.3-8.1)	< 0.001
Mean duration from disease onset to effective antibiotic therapy (days)	8.34 ± 5.1	7.11 ± 3.7		0.1
				
Underlying diseases (Diabetes, Liver cirrhosis, COPD)	6 (6.8%)	4 (3.3%)	2.1(0.6-7.8)	0.25
Headache	74 (83.1)	109 (91.6)	0.5 (0.2-1.1)	0.07
Myalgia	66 (74.2)	95(79.8)	0.7(0.4-1.4)	0.3
Skin rash	78(87.6)	111 (93.3)	0.5 (0.2-1.3)	0.2
Eschar	77 (86.5)	117 (98.3)	0.1 (0.02-0.5)	0.004
WBC count (> 10, 000/mm^3^)	37 (41.6%)	16 (13.4%)	4.6 (2.3-9.0)	< 0.001
Hemoglobin (≤ 10 g/dL)	10 (11.2%)	2 (1.7%)	0.14(0.03-0.63)	0.01
Platelet count (≤ 100, 000/mm^3^)	28(31.5%)	25(21.0%)	0.58(0.31-1.09)	0.09
AST (>40 IU)	79(88.8%)	108 (90.8%)	1.24(0.50-3.07)	0.64
ALT (>40 IU)	84(94.4%)	106 (89.1%)	1.0 (1.0-1.0)	0.16
ALP (>200 U/L)	19(21.6%)	17 (14.9%)	1.6(0.79-3.3)	0.19
Bilirubin (>1.0 mg/dL)	23(25.8%)	22 (18.5%)	1.54(0.79-2.98)	0.2
Albumin (≤ 3.0 g/dL)	28(31.5%)	6(5.1%)	8.5(3.32-21.64)	> 0.001
LDH (>800 U/L)	37(43.0%)	63(53.8%)	0.65(0.37-1.13)	0.13
CPK (>200 U/L)	24(27.9%)	31(26.7%)	1.06(0.57-1.98)	0.85
ADA (<100 IU/L)	13(22.0%)	13(19.4%)	1.17(0.50-2.78)	0.72
Serum creatinine (> 1.4 mg/dL)	31(34.8%)	3(2.5%)	20.7(6.06-70.4)	< 0.001
Fibrinogen (≤ 150 mg/dL)	6(7.2%)	3(2.8%)	0.37(0.09-1.51)	0.17
CRP (> 10 mg/dL)	37(44.6%)	29(24.8%)	2.44(1.34-4.46)	0.004
ESR (> 20 mm/hr)	33(39.8%)	41(35.3%)	1.21(0.68-2.16)	0.53
PT (> 15 sec)	2(2.2%)	2(1.7%)	1.31(0.18-9.49)	0.79
aPTT (>40 sec)	9(10.1%)	4 (3.4%)	3.15(0.94-10.59)	0.06

Abbreviations as in Table 1

Multivariate analysis demonstrated that only the following four factors were significantly associated with the severe scrub typhus (Table 4): (1) age ≥ 60 years (OR = 3.13, *P *= 0.002, CI = 1.53-6.41), (2) the absence of eschar (OR = 6.62, *P *= 0.03, CI = 1.22-35.8), (3) WBC counts > 10, 000/mm^3 ^(OR = 3.6, *P *= 0.001, CI = 1.65-7.89), and (4) albumin ≤ 3.0 g/dL (OR = 5.01, *P *= 0.004, CI = 1.69-14.86).

**Table 4:** Unadjusted and adjusted relative risk of severe scrub typhus *

Factors	Unadjusted Odds ratio	Adjusted		*P *value
		Odds ratio	95% CI	
Age(year) >60	4.3	3.13	1.53-6.41	0.002
Abscence of Eschar	9.1	6.62	1.22-35.8	0.03
WBC count (> 10, 000/mm^3^)	4.6	3.6	1.65-7.89	0.001
Albumin(≤ 3.0 g/dL)	8.5	5.01	1.69-14.86	0.004

*Only significant risk factors in Table 2 except creatinine were selected for the analysis

Scrub typhus is a noticeable mite-borne disease with its incidence being highest in Korea [11]. There have so far been a few reports on severe complications and the mortality rate of scrub typhus [8]. Varghese et al [8] have reported that in 50 patients with the elevated IgM antibody who presented with fever in the southern part of India, the elevated serum creatinine level is an independent predictor for fatal outcome of scrub typhus patients. However, in their study, age, albumin, the absence or presence of eschar were not evaluated, and its sample size was too small to determine the risk factors.

In this study, by multivariate analysis, age ≥ 60 years, the absence of eschar, WBC counts > 10, 000/mm^3^, and albumin ≤ 3.0 g/dL were found to be independently predictive variables for the occurrence of severe scrub typhus.

The occurrence rates of eschar are reported to differ among regions [12]. Eschar could be detected relatively frequently in reports in Japan and Korea [12,13], but relatively less in Thailand and Taiwan [14,15].

In our study, mean duration from disease onset to effective antibiotic therapy (days) is not different between the scrub typhus patients with and without severe severity. Assuming that the hospital visit is delayed according to absence of rash or eschar, the length of time from the onset of clinical symptoms to the initial hospital visit was analyzed in the patients with eschar and those without eschar. In this analysis, the length of time from the onset of clinical symptoms to the hospital visit was 10.43 ± 6.442 days in the patients without eschar and 7.24 ± 4.32 days in those with eschar, and the difference was statistically significant (data not shown, *P *= 0.011). Most scrub typhus patients with eschar are presented with rash in our study. Patients with rash or eschar tend to go see a doctor earlier in the disease course. In a Mediterranean spotted fever report [16], a fatal outcome was significantly more likely for patients infected with the ISF strain than Malish strain. Eschar was observed in a significantly higher percentage of patients infected with the Malish strain (60%), compared with patients infected with the ISF strain (38%). In that study, even though ISF strain infected patients received effective antirickettsial treatment earlier in the course of the disease, fatality was much higher. In scrub typhus patients, there is a possibility of the differences in severity or clinical features like rash or eschar according to the serotypes or genotypes of *O. tsutsugamushi*; It is possible that scrub typhus patients contracted with more virulent strain have lower incidence of rash or eschar. Large scale studies are needed to confirm the difference in virulence according to their genotypes or serotypes.

Varghese et al [8] reported that median age of scrub typhus patients in the southern part of India was 36.5 (range: 12-75 years), and the mortality rate was 14%. In one study from Taiwan, Lai CH et al [14] reported that mean age of scrub typhus patients was around 43. Watt G et al [17] reported the case fatality rate of 15% ~30% in scrub typhus patients from Thailand. However, in our study, only one patient died of scrub typhus and there was no one who needed ventilation support or renal replacement therapy. In Korea the population of the farming villages in rural areas is getting old just like one study from Japan [2,11], the greatest number of scrub typhus patients in Korea was in the age group 50-69 years. Many papers reporting scrub typhus in Korea have presented mortality much less than 10% [18]. Despite the fact that this study included older patients than other studies, the mortality rate was relatively lower. One plausible explanation for this result is that our patients visited the hospital earlier, were diagnosed with scrub typhus at the early stage, and subsequently received appropriate treatment quickly because more than 90% of our patients had an eschar or rash. Another explanation is that scrub typhus patients enrolled in our study were infected with *O. tsutsugamushi *of relatively lower virulence. However, further studies are needed to confirm the relationship between the presence of eschar and the mortality rate. In addition to the presence of eschar, WBC counts were significantly higher in the severe scrub typhus group, suggesting that this group was more seriously infected and was accompanied by more severe inflammation.

In this study, 16.3% of patients with a definitive diagnosis of scrub typhus showed albumin ≤ 3.0 g/dL, and the serum albumin level decreased significantly in the severe scrub typhus group (31.5% in the severe scrub typhus group vs. 5.1% in the control group; *P *< 0.001). Although the pathogenetic mechanisms for hypoalbuminemia in patients with scrub typhus have not yet been elucidated, it is postulated that vasculitis, the main pathology of scrub typhus, increases vascular permeability, and thus plasma protein leaked from the blood vessels with subsequently leading to hypoalbuminemia[19]. Min et al [20] have demonstrated that patients with scrub typhus showed intestinal protein loss by measuring the concentrations of α-1-antitrypsin in the stool, and that 11 of 14 patients with scrub typhus revealed intestinal albumin loss by ^99m^Tc-human serum albumin (HSA) abdominal scintigraphy. The presumed mechanism for the intestinal albumin loss is the increase in permeability due to vasculitis of the capillaries and arterioles [20]. It is well known that in patients with sepsis, hypoalbuminemia has a significant correlation with the severity and mortality rate of the disease, and that the mortality of patients with sepsis become higher with the increasing severity of hypoalbuminemia [21]. Because hypoalbuminemia could be a complication of liver cirrhosis, we reassessed the serum albumin level excluding the patients with liver cirrhosis; But serum albumin level was still decreased significantly in the severe scrub typhus group (data not shown).

There are some limitations in this study. First, we could not ascess the mortality risk factors because of lack of the number of deceased patients. Second, we could not evaluate the differences in severity or mortality or difference in virulence according to genotypes or serotypes of *O. tsutsugamushi*. Third, only hospitalized patients were included. Fourth, the diagnosis of scrub typhus was based on serologic analysis alone, not by PCR or culture. Finally, the application of our results to other regions may be limited because of the geographic differences in prevailing serotype;In Korea, most of them is Boryong serotype a with high chance of eschar formation.

Nevertheless, these four parameters suggested in our study are simple and can be checked in most hospitals, even in developing countries. The presence of one or more of these four risk factors for severe scrub typhus should alert physicians to provide close monitoring and intensive care, which can prevent severe morbidity or mortality.

## Conclusions

In summary, the results of this study suggest that clinicians should be aware of the potential for severe scrub typhus, when scrub typhus patients are older (≥ 60 years), present with the absence of eschar, or laboratory findings such as WBC counts > 10, 000/mm^3^, and serum albumin level ≤ 3.0 g/dL. Close observation and intensive care for scrub typhus patients with the potential for severe manifestations may prevent severe morbidity or mortality.

## Competing interests

The authors declare that they have no competing interests.

## Authors' contributions

DK made a substantial contribution to draft the manuscript and revised the draft all over the course of submission. SC and NY participated in revision of the manuscript. SK conceived of the study, participated in its design and coordination and drafted the manuscript. All authors read and approved the final manuscript.

## Pre-publication history

The pre-publication history for this paper can be accessed here:

http://www.biomedcentral.com/1471-2334/10/108/prepub

## References

[B1] Yi KS Chong Y Covington SC Donahue BJ Rothen RL Rodriguez J Arthur JD Scrub typhus in Korea: importance of early clinical diagnosis in this newly recognized endemic area Mil Med 1993 158 269 73 8479637

[B2] Ogawa M Hagiwara T Kishimoto T Shiga S Yoshida Y Furuya Y Kaiho I Ito T Nemoto H Yamamoto N Masukawa K Scrub typhus in Japan: epidemiology and clinical features of cases reported in 1998 Am J Trop Med Hyg 2002 67 162 5 12389941 10.4269/ajtmh.2002.67.162

[B3] Kim SJ, IK Chung IS Chung DH Song SH Park HS Kim MH The clinical significance of upper gastrointestinal endoscopy in gastrointestinal vasculitis related to scrub typhus Endoscopy 2000 32 950 5 10.1055/s-2000-9621 11147943

[B4] Thap LC Supanaranond W Treeprasertsuk S Kitvatanachai S Chinprasatsak S Phonrat B Septic shock secondary to scrub typhus: characteristics and complications Southeast Asian J Trop Med Public Health 2002 33 780 6 12757226

[B5] Yen TH Chang CT Lin JL Jiang JR Lee KF Scrub typhus: a frequently overlooked cause of acute renal failure Ren Fail 2003 25 397 410 10.1081/JDI-120021152 12803503

[B6] Tsay RW Chang FY Serious complications in scrub typhus J Microbiol Immunol Infect 1998 31 240 4 10496165

[B7] Hu ML Liu JW Wu KL Lu SN Chiou SS Kuo CH Chuah SK Wang JH Hu TH Chiu KW Lee CM Changchien CS Short report: Abnormal liver function in scrub typhus Am J Trop Med Hyg 2005 73 667 8 16222006

[B8] Varghese GM Abraham OC Mathai D Thomas K Aaron R Kavitha ML Mathi E Scrub typhus among hospitalised patients with febrile illness in South India: magnitude and clinical predictors J Infect 2006 52 56 60 10.1016/j.jinf.2005.02.001 16368461

[B9] World Health Organization (WHO) WHO recommended surveillance standards 2004 2 http://www.who.int/csr/resources/publications/surveillance/whocdscsrisr992.pdf

[B10] Klawansky S Komaroff E Cavanaugh PF Jr Mitchell DY Gordon MJ Connelly JE Ross SD Relationship between age, renal function and bone mineral density in the US population Osteoporos Int 2003 14 570 6 10.1007/s00198-003-1435-y 12844211

[B11] Kweon SS Choi JS Lim HS Kim JR Kim KY Ryu SY Yoo HS Park O Rapid increase of scrub typhus, South Korea, 2001-2006 Emerg Infect Dis 2009 15 1127 9 10.3201/eid1507.080399 19624938 PMC2744253

[B12] Kim DM Won KJ Park CY Yu KD Kim HS Yang TY Lee JH Kim HK Song HJ Lee SH Shin H Distribution of eschars on the body of scrub typhus patients: a prospective study Am J Trop Med Hyg 2007 76 806 9 17488895

[B13] Silpapojakul K Chupuppakarn S Yuthasompob S Varachit B Chaipak D Borkerd T Silpapojakul K Scrub and murine typhus in children with obscure fever in the tropics Ped Infect Dis J 1991 10 200 3 10.1097/00006454-199103000-00006 2041666

[B14] Lai CH Huang CK Weng HC Chung HC Liang SH Lin JN Lin CW Hsu CY Lin HH The difference in clinical characteristics between acute Q fever and scrub typhus in southern Taiwan Int J Infect Dis 2009 13 387 93 10.1016/j.ijid.2008.07.020 18977677

[B15] Charoensak A Chawalparit O Suttinont C Niwattayakul K Losuwanaluk K Silpasakorn S Suputtamongkol Y Scrub typhus: chest radiographic and clinical findings in 130 Thai patients J Med Assoc Thai 2006 89 600 7 16756043

[B16] Sousa R França A Dória Nòbrega S Belo A Amaro M Abreu T Poças J Proença P Vaz J Torgal J Bacellar F Ismail N Walker DH Host- and microbe-related risk factors for and pathophysiology of fatal Rickettsia conorii infection in Portuguese patients J Infect Dis 2008 198 576 85 10.1086/590211 18582199 PMC2614375

[B17] Watt G Kantipong P Jongsakul K Watcharapichat P Phulsuksombati D Strickman D Doxycycline and rifampicin for mild scrub-typhus infections in Northern Thailand: a randomised trial Lancet 2000 356 1057 61 10.1016/S0140-6736(00)02728-8 11009140

[B18] Wie SH Chang UI Kim HW Hur J Kim SI Kim YR Kang MW Clinical features of 212 cases of scrub typhus in southern region of Gyeonggi-do and the significance of initial simple chest x-ray Infect Chemother 2008 40 40 5 10.3947/ic.2008.40.1.40

[B19] Kim YO Jeon HK Cho SG Yoon SA Son HS Oh SH Chae HS Kim KH Chae JS Lee CD Lee BS The role of hypoalbuminemia as a marker of the severity of disease in patients with tsutsugamushi disease Korean J Internal Med 2000 59 516 21

[B20] Min JK Jung WC Baek GH Kim YR Oh SH Kang MW Chung IS Yang WJ Kim SH Intestinal Protein Loss in Patients with Tsutsugamushi Disease Korean J Internal Med 1996 51 457 64

[B21] Goldwasser P Felman J Association of serum albumin and mortality risk J Clin Epidemiol 1997 50 6 693 703 10.1016/S0895-4356(97)00015-2 9250267

